# Comparison between microendoscopic laminectomy and open posterior decompression surgery for single-level lumbar spinal stenosis: a multicenter retrospective cohort study

**DOI:** 10.1186/s12891-021-04963-6

**Published:** 2021-12-20

**Authors:** Nozomu Ohtomo, Hideki Nakamoto, Junya Miyahara, Yuichi Yoshida, Hiroyuki Nakarai, Keiichiro Tozawa, Masayoshi Fukushima, So Kato, Toru Doi, Yuki Taniguchi, Yoshitaka Matsubayashi, Akiro Higashikawa, Yujiro Takeshita, Naohiro Kawamura, Hirohiko Inanami, Sakae Tanaka, Yasushi Oshima

**Affiliations:** 1 Department of Orthopaedic Surgery, The University of Tokyo Hospital, 7-3-1Bunkyo-Ku, HongoTokyo, 113-8655 Japan; 2 University of Tokyo Spine Group (UTSG), 7-3-1Bunkyo-Ku, HongoTokyo, 113-8655 Japan; 3 Department of Spine and Orthopedic Surgery, Japanese Red Cross Medical Center, 4-1-22Shibuya-Ku, HirooTokyo, 150-8935 Japan; 4 Department of Orthopedic Surgery, Kanto Rosai Hospital, 1-1, Kizukisumiyoshi-Cho, Nakahaha-Ku, Kawasaki City, Kanagawa, 211-8510 Japan; 5 Department of Orthopedic Surgery, Yokohama Rosai Hospital, 3211 Kozukue-Cho, Kohoku-Ku, Yokohama City, Kanagawa, 222-0036 Japan; 6 Spine Center, Toranomon Hospital, 2-2-2Minato-Ku, ToranomonTokyo, 105-8470 Japan; 7 Inanami Spine and Joint Hospital, 3-17-5Shinagawa-Ku, HigashishinagawaTokyo, 140-0002 Japan

**Keywords:** Multicenter retrospective cohort study, Microendoscopic laminectomy, Patient-reported outcomes, Complications

## Abstract

**Background:**

Microendoscopic laminectomy (MEL), in which a 16-mm tubular retractor with an internal scope is used, has shown excellent surgical results for patients with lumbar spinal canal stenosis. However, no reports have directly compared MEL with open laminectomy. This study aimed to elucidate patient-reported outcomes (PROs) and perioperative complications in patients undergoing MEL versus open laminectomy.

**Methods:**

This is a multicenter retrospective cohort study of prospectively registered patients who underwent lumbar spinal surgery at one of the six high-volume spine centers between April 2017 and September 2018. A total of 258 patients who underwent single posterior lumbar decompression at L4/L5 were enrolled in the study. With regard to demographic data, we prospectively used chart sheets to evaluate the diagnosis, operative procedure, operation time, estimated blood loss, and complications. The follow-up period was 1-year. PROs included a numerical rating scale (NRS) for lower back pain and leg pain, the Oswestry Disability Index (ODI), EuroQol 5 Dimension (EQ-5D), and patient satisfaction with the treatment.

**Results:**

Of the 258 patients enrolled, 252 (97%) completed the 1-year follow-up. Of the 252, 130 underwent MEL (MEL group) and 122 underwent open decompression (open group). The MEL group required a significantly shorter operating time and sustained lesser intraoperative blood loss compared with the open group. The MEL group showed shorter length of postoperative hospitalization than the open group. The overall complication rate was similar (8.2% in the MEL group versus 7.7% in the open group), and the revision rate did not significantly differ. As for PROs, both preoperative and postoperative values did not significantly differ between the two groups. However, the satisfaction rate was higher in the MEL group (74%) than in the open group (53%) (p = 0.02).

**Conclusions:**

MEL required a significantly shorter operating time and resulted in lesser intraoperative blood loss compared with laminectomy. Postoperative PROs and complication rates were not significantly different between the procedures, although MEL demonstrated a better satisfaction rate.

## Background

Degenerative lumbar diseases are accompanied by lower back pain, leg pain, or neurogenic intermittent claudication, all of which affect the patients’ health-related quality of life. Surgical intervention is often considered in those patients with longstanding symptoms that are unresponsive to conservative treatment. Surgical interventions include decompression and decompression with fusion. In patients with spondylolisthesis or foraminal stenosis, fusion surgery is required. Otherwise, decompression surgery is indicated for most cases with central spinal stenosis or with radiculopathy resulting from lateral recess stenosis [1–7]. In decompression surgery, laminectomy and flavectomy are usually performed at the spinal level causing the symptoms. Classically, the spinous process and interspinous muscles are removed to expose the lamina; however, this might lead to the development of lower back pain or segmental instability. To overcome these surgery-related problems, several less-invasive techniques have been proposed, one of which is microendoscopic laminotomy (MEL) [8]. This technique, in which a 16-mm tubular retractor with an internal scope is used, was originally applied in cases of lumbar disc herniation. Bilateral decompression via a unilateral approach is performed for cases with central canal stenosis, and several reports on the efficacy of MEL for the treatment of lumbar spinal canal stenosis have been published [9–11].　However, few reports have directly compared surgical outcomes between MEL and open laminectomy.

One possible problem in the comparison of surgical outcomes of MEL with open surgical techniques is the heterogeneity of patients in each procedure. Moreover, MEL is a precise technique with a relatively small bony resection margin and a favorable indication for single-level cases. Thus, a surgeon who is skilled in microendoscopic techniques would still prefer open surgery for cases with multilevel canal stenosis. Therefore, we sought to obtain data from a homogeneous population by conducting a multicenter cohort study focusing on patients with single-level spinal canal stenosis of the lumbar spine (L4/L5). This study compared patient-reported outcomes (PROs) and perioperative complications between patients with lumbar spinal canal stenosis undergoing MEL and those undergoing open laminectomy.

## Methods

We prospectively analyzed registered patients who underwent lumbar spinal surgery at one of the six high-volume spine centers between April 2017 and September 2018. Patients with a history of previous lumbar surgery, tumor, infection, rheumatoid arthritis, or deformity were excluded from this study. This study was approved by the Institutional Review Boards of each of the five hospitals. Written informed consent was obtained from all participants.

**Fig. 1:**
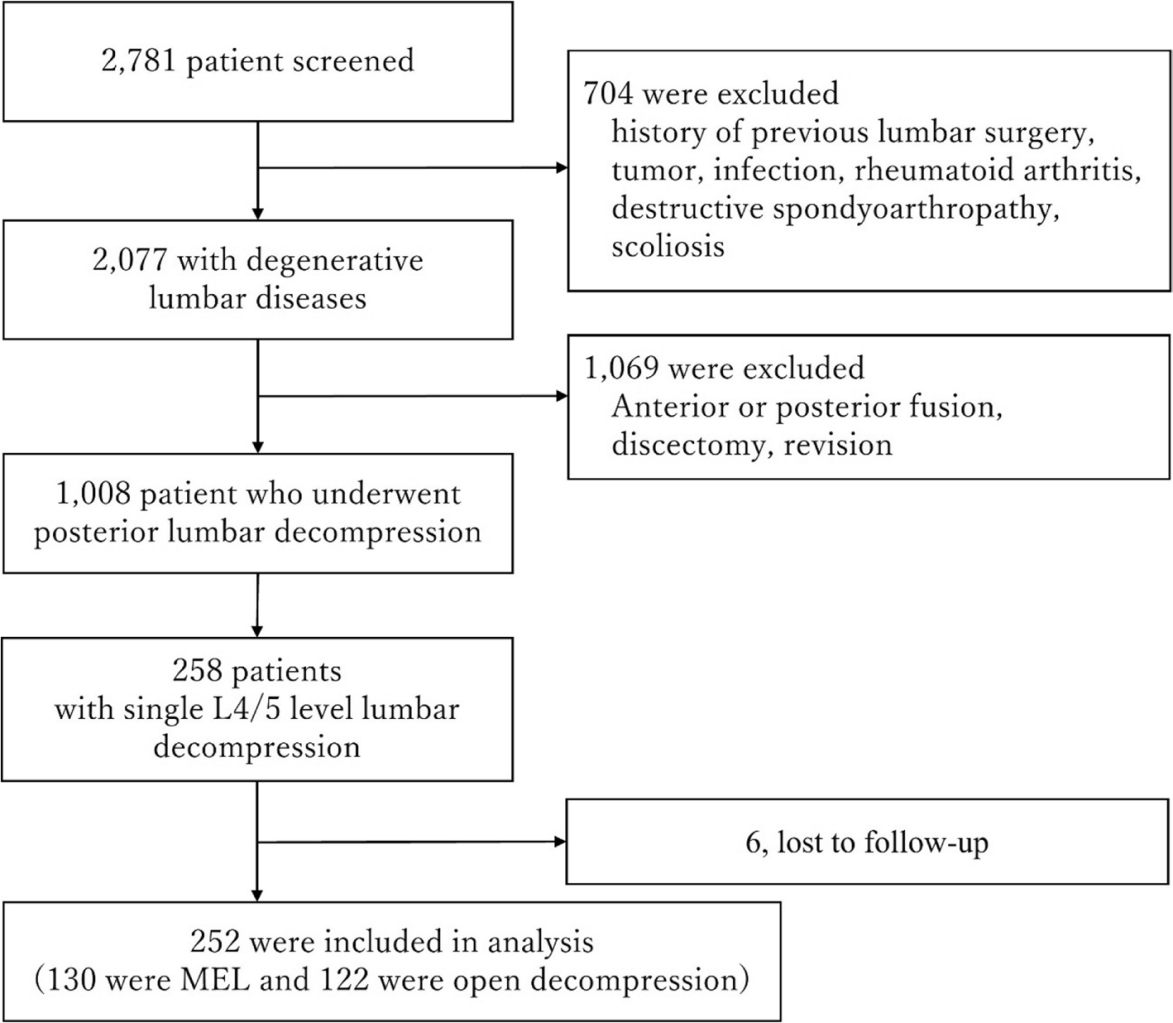
Flow diagram of the selection of study participants

A total of 2,781 patients were included in the study (Fig. 1). After applying the exclusion criteria, 1,008 patients who underwent posterior decompression surgery were finally. Of them, 258 underwent single posterior lumbar decompression at L4/L5 and were subsequently enrolled for further analysis. Patients were required to complete both preoperative and postoperative (1-year) PROs.

Surgical strategy was determined at each hospital. One facility only performed MEL on all patients, whereas the other five performed both MEL and open procedures. MEL was performed using the METRx system (Medtronic Sofamor Danek) as previously described [12]. Briefly, a 20-mm skin incision was made 1 cm off the midline, and a 16-mm tubular retractor with an internal scope was inserted into the lamina. Ipsilateral laminectomy and removal of the yellow ligament were performed for radiculopathy cases. For cases requiring bilateral decompression, laminotomy of the contralateral side was performed in addition (bilateral decompression via a unilateral approach). As for open laminectomy, partial laminectomy was performed using a midline approach, the details of which depended on the surgeon. The same kind of tubular retractors were not used in the open techniques.

With regard to the demographic data, we prospectively used the chart sheets to collect data on diagnosis, operative procedure, operation time, estimated blood loss, and complications. The PROs included a numerical rating scale (NRS) for lower back pain and leg pain, Oswestry Disability Index (ODI), EuroQol 5 Dimension (EQ-5D), and an assessment of patient satisfaction with the treatment. A 7-point Likert scale was utilized to determine patient satisfaction, with possible answers including “very dissatisfied,” “dissatisfied,” “somewhat dissatisfied,” “unsure,” “somewhat satisfied,” “satisfied,” and “very satisfied.” We defined the patients who answered “somewhat satisfied,” “satisfied,” or “very satisfied” as “satisfactory.”

Statistical evaluation was conducted using the JMP software version 12.0 (SAS Institute Inc., Cary, North Carolina, USA). The Mann–Whitney U test was employed to analyze nonparametric data, and the chi-squared or Fisher’s exact test was used to analyze categorical variables. A p-value of < 0.05 was considered statistically significant.

## Results

Of the 258 patients enrolled, 252 (97%) completed the 1-year follow-up. Of these patients, 130 underwent MEL (MEL group) and 122 underwent open decompression (open group). One facility exclusively performed MEL on all patients with single-level disease, whereas the other five performed both procedures.

The demographic data for each group are listed in Table 1. No significant differences were observed in the baseline characteristics between the two groups. The findings presented in Table 2 include a comparison of perioperative data between the MEL and open groups. The MEL group required a significantly shorter operating time and incurred lesser intraoperative blood loss than the open group. The length of postoperative hospitalization was shorter in the MEL group (9.7 days) than the open group (16.5 days) (p = 0.02). The overall complication rate was similar for both groups (8.2% in the MEL group versus 7.7% in the open group), whereas the revision rate did not significantly differ. However, the features of complications varied; dural tears were more often observed in the MEL group, whereas surgical site infections (SSI) were more common in the open group, although the differences were not significant. No patient had L5 paralysis after surgery in both groups.

**Table 1:** Demographic data of the patients in microendoscopic laminectomy and open groups

	**MEL Group**	**Open Group**	**p**
Mean age (years, range)	74.6 (39–88)	73.8 (38–88)	0.38
Gender (M/F)	69/61	68/54	0.92
Average height (cm, range)	156.9 (135.0–180.3)	159.6 (141.0–179.5)	0.95
Average body weight (kg, range)	60.2 (32.9–101.0)	61.8 (41.2–97.6)	0.34
BMI (range)	24.1(18.0–34.9)	24.8 (18.1–31.3)	0.52
Type of spinal canal stenosis (Central/lateral recess)	58/64	55/62	0.94

*MEL*, microendoscopic laminectomy, *BMI*, body mass index

**Table 2:** Comparison of operative data between microendoscopic laminectomy and open groups

	**MEL Group**	**Open Group**	**p**
Operative time (min, range)	76.8 (40–169)	100.9 (47–274)	0.04
Intraoperative blood loss (mL, range)	30.6 (5–250)	144.2(75–700)	0.001
Postoperative hospitalization (days)	9.7	16.5	0.02
Complications (%)			
Total	10 (8.2)	9 (7.7)	0.88
Dual tear	8 (6.5)	3 (2.5)	0.21
SSI	1 (0.8)	4 (3.4)	0.16
Hematoma	1 (0.8)	0	0.32
UTI	0	1 (0.8)	0.30
Stroke	0	1 (0.8)	0.30
Revision (%)	1: hematoma (0.8)	2: SSI (1.7)	0.97

*MEL*, microendoscopic laminectomy, *SSI*, surgical site infections, *UTI*, urinary tract infections

As for PROs, both preoperative and postoperative values did not significantly differ between the two groups (Table 3). However, the satisfaction rate was higher in the MEL group (74%) than in the open group (53%) (p = 0.02).

**Table 3:** Comparison of patient-reported outcomes between microendoscopic laminectomy and open groups

	**MEL Group**	**Open Group**	**p**
**NRS (Lower back pain)**			
Preoperative	5.92 ± 2.6	5.96 ± 2.8	0.21
Postoperative	2.43 ± 1.5	2.67 ± 1.2	0.72
**NRS (Leg pain)**			
Preoperative	5.80 ± 2.7	5.72 ± 3.4	0.76
Postoperative	2.52 ± 1.6	2.36 ± 2.4	0.98
**ODI**			
Preoperative	20.8 ± 8.2	20.2 ± 9.7	0.80
Postoperative	12.0 ± 5.8	11.8 ± 6.2	0.34
**EQ-5D**			
Preoperative	0.58 ± 0.12	0.55 ± 0.18	0.78
Postoperative	0.74 ± 0.17	0.73 ± 0.12	0.98
**Satisfaction with the operation**	74%	53%	0.02

*MEL*, microendoscopic laminectomy, *NRS*, numerical rating scale, *ODI*, Oswestry Disability Index, *EQ-5D*, EuroQol 5 Dimension

## Discussion

We sought to compare the PROs and surgery-related complications between MEL and open decompression in patients with single-level lumbar spinal stenosis and found no significant between-group differences in either PROs or complication rates. To the best of our knowledge, this is the first report that directly compares the PROs between MEL and open laminectomy.

Microendoscopic decompression surgery has been primarily developed for herniated discs or spinal canal stenosis of the lumbar spine [13, 14]. The initial concept of this technique was hemilaminectomy using a paramedian approach approximately 1 cm off the midline using a 16- or 18-mm tubular retractor with an internal scope. It was indicated for unilateral radiculopathy cases with lumbar disc hernia or lateral recess stenosis. This technique was subsequently developed for cases with central canal stenosis that require bilateral decompression by adding laminotomy of the contralateral side. Favorable surgical outcomes have been obtained with MEL, as previously demonstrated by several reports [15–17]. Minamide et al. investigated surgical outcomes in 310 cases following MEL 2 years postoperatively and reported a Japanese Orthopaedic Association score recovery rate of 61.3% and Roland–Morris Disability Questionnaire scores that significantly improved from 11.3 to 4.8, although it was a single-arm study and did not compare MEL with open procedures. Only one report directly compared the complication rates between MEL and open laminectomy in patients with lumbar spinal stenosis. [18] This study, which used a national database in Japan to identify 1,536 MEL cases (6.6%) out of a total of 23,317 laminectomy cases, demonstrated a lower incidence of SSI (0.5% in MEL versus 1.6% in open laminectomy) or major complications, such as pulmonary embolism and brain infarction (1.0% in MEL versus 2.8% in open laminectomy) after adjustment by propensity score matching. They succeeded in demonstrating the minimal invasiveness of MEL over the open techniques; however, they did not investigate the PROs.

Although we could not find any reports that directly compared PROs between MEL and open decompression surgery for lumbar spinal canal stenosis, several past reports have indicated the advantages of using a tubular retractor under a microscopic view instead of microendoscopic usage. According to a systematic review and meta-analysis that compared surgical outcomes between minimally invasive unilateral laminectomy for bilateral decompression (ULBD) and standard open laminectomy, ULBD resulted in reduced postoperative lower back pain, superior satisfaction rates, reduced hospitalization, and less blood loss but required a longer operating time [19]. Because the concept of MEL is basically the same as that of tubular surgeries without a microendoscope, we anticipated that the severity of postoperative lower back pain at 1 year would be lower in the MEL group. In the present study, however, we did not find any significant between-group differences in the PROs at 1 year after surgery. We speculate that this was because the open laminectomies used in this series did not require extensive exploration of the posterior supporting structures. Nevertheless, MEL had a better patient satisfaction rate. The minimal invasiveness of MEL, as indicated by lesser blood loss and a shorter operating time, would have led to reduced perioperative pain and a shorter period of postoperative bed rest, thereby achieving a higher satisfaction rate. This reasonable speculation is in agreement with the findings of a previous report, which described a higher satisfaction rate for cervical microendoscopic decompression compared with laminoplasty [20].

One concern of using a microendoscope is the steep learning curve required [21]. Indeed, controversies have arisen over whether MIS techniques have a higher incidence of surgery-related complications. Of note, surgeons pay more attention to avoiding complications such as dural tears, nerve injuries, vessel injuries, or implant malpositioning in performing MIS spine surgery. Although no previous reports directly compared surgery-related complications between lumbar MEL and open laminectomy, several reports have demonstrated the incidence and prognosis of the cases with complications following MEL. Minamide et al. [17] reported 12 surgery-related complications out of 310 MEL cases, including dural tears (1.9%), wrong-level operations (0.3%), transient neuralgias (1.3%), and infections (0.3%), although all of them fully recovered spontaneously. Soma et al. [22] investigated the influence of dural tears following lumbar MEL in 922 patients and found that 49 (5.3%) of them suffered from dural tear and 23 (2.5%) required suture repair. However, postoperative PROs were not influenced. They speculated that reduced muscle trauma in MEL prevented severe postoperative headaches or revision surgeries. In the current study, the incidence of dural tears was not statistically significant between the two groups, although the incidence of dural tears tended to be higher in MEL cases. Considering that no patients required revision surgery for dural tears, they were adequately treated by microendoscopy as previously described [22]..

One thing that should be noted is the likelihood that more experienced surgeons tended to perform MEL than open laminectomy in the current study. Moreover, surgeons might have preferred open techniques in patients with spinal stenosis at L3/L4 or L2/L3 as the unilateral approach could impair facet joints, which are located inside at the upper lumbar levels, compared with stenosis at L4/L5. To eliminate surgeon’s bias in deciding the surgical procedure, we focused on single-level cases involving L4/L5, at which spinal stenosis is most commonly observed. Nevertheless, we consider that the surgery-related complication rate of microendoscopic surgery is not significantly higher than open techniques if it is adequately indicated.

Water-medium spinal endoscopy, which has become more popular in recent years, is rather suitable for the decompression of small areas, namely, radiculopathy [23, 24]. Conversely, for cases with central canal stenosis in which wider bony resection and yellow ligament removal is required, tubular surgery under air medium can provide a wider view of the surgical field than the full endoscopic approach with a narrower retractor. In the current study, we encountered no cases in which ULBD was performed using a tubular retractor under microscopic visualization instead of a microendoscopic one. Although we speculate that the results would not have significantly differed, MEL provides a better surgical field of view free of obstruction by the surgeon’s hands. Moreover, the tubular retractor is more freely movable like a joystick without the need to readjust for visualization, which is often required in microscopic surgery. Therefore, we believe that MEL remains as one of the best surgical procedures, particularly for central canal stenosis.

There are several limitations to this study. First, due to the multicenter scope of the study, the differences in surgical indications, surgeons’ skill, procedures and postoperative rehabilitation may have had an unanticipated impact on the results, and also, owing to the retrospective nature of this study, the existence of slip or scoliosis was not examined. However, as the preoperative PROs did not significantly differ between the MEL and open groups, we presume no significant selection biases exist. Second, this study did not examine the severity of spinal stenosis at L4/5 on MRI, which might have affected the PROs. Third, the follow-up period was only for 1 year. A longer follow-up interval may yield different results, although the PROs evaluated at 1 year postoperatively can be indicative of longer-term prognosis (24 months) [25]. Finally, this study only focused on single-level cases at L4/L5 to ensure a homogeneous population. Therefore, the results may not be generalizable to multilevel cases. Regardless of these limitations, we believe the results of our study contribute contemporary data that will help inform surgical management decisions.

## Conclusions

Our findings demonstrate that MEL has a significantly shorter operating time and lesser intraoperative blood loss than open laminectomy. The MEL group showed shorter length of postoperative hospitalization than the open group. Postoperative PROs and complication rates did not differ significantly, although MEL achieved a better patient satisfaction rate.

## Data Availability

The datasets generated and/or analyzed during the current study are not publicly available due to their containing information that could compromise the privacy of research participants but are available from the corresponding author on reasonable request.

## Abbreviations

MEL: Microendoscopic laminotomy
PROs: Patient-reported outcomes
ODI: Oswestry Disability Index
EQ-5D: EuroQol 5 Dimension
